# The Readability of Patient Education Materials Pertaining to Gastrointestinal Procedures

**DOI:** 10.1155/2021/7532905

**Published:** 2021-04-02

**Authors:** Mohammad S. Nawaz, Laura E. McDermott, Savanna Thor

**Affiliations:** ^1^Department of Gastroenterology & Hepatology, SUNY Downstate Health Sciences University, Brooklyn 11203, New York, NY, USA; ^2^Department of Cardiology, Jackson Memorial Hospital, University of Miami, Miami 33136, FL, USA

## Abstract

**Introduction:**

Due to the ubiquity and ease of access of Internet, patients are able to access online health information more easily than ever. The American Medical Association recommends that patient education materials be targeted at or below the 6^th^ grade level in order to accommodate a wider audience. In this study, we evaluate the difficulty of educational materials pertaining to common GI procedures; we analyze on the readability of online education materials for colonoscopy, flexible sigmoidoscopy, and esophagogastroduodenoscopy (EGD).

**Methods:**

Google search was performed using keywords of “colonoscopy,” “sigmoidoscopy,” and “EGD” with “patient information” at the end of each search term. The texts from a total of 18 studies, 6 for each of the procedures, were then saved. Each study was also subdivided into “Introduction,” “Preparation,” “Complications,” and if available, “Alternatives.” Furthermore, medical terminology that was properly explained, proper nouns, medication names, and other copyright text were removed in order to prevent inflation of the difficulty. Five validated readability tests were used to analyze each study and subsections: Coleman-Liau, New Dale-Chall, Flesch-Kincaid, Gunning Fog, SMOG.

**Results:**

Studies on colonoscopy, flexible sigmoidoscopy, and EGD had median readability grades of 9.7, 10.2, and 11.0, respectively. Analysis of the subsections revealed that the “Alternative” subsection was the most difficult to comprehend with a readability score of 11.4, whereas the “Introduction” subsection was the easiest to comprehend with a readability score of 9.5.

**Conclusion:**

Despite modifications to the studies that improved the readability scores, patient education materials were still significantly above the recommended 6^th^ grade level across all websites. This study emphasizes that clear and simple language is warranted in order to create information that is suitable for most patients.

## 1. Introduction

With the ubiquity of Internet and its availability on many different devices, access to information has become much easier than ever. Pew Research Center reported in 2013 that 59% of adults use Internet to search for health information [1]. Majority of them used search engines, such as Google and Bing, to initiate the search. According to the study, the younger population under the age of 35 was more likely to use Internet (close to 80%) for seeking medical information. Online health information also has the potential to influence health behaviors, as one-third of those under the age of 30 years adjusted the way they took care of themselves based on the information they obtained [2]. It can be argued that Internet has an impact in raising peoples' awareness on their health. With such accessibility, the quality of the written material comes into question. A study published in the Journal of Pediatrics examining online information on infant sleep position showed that only 43.5% of 1300 websites examined provided accurate information [3]. Another aspect behind quality patient education materials is literature that is written at a level that can be comprehended by most adults.

Readability is the ease at which an audience can comprehend text. By many metrics, this is reported as a grade level. For example, text with a readability of 10 means it can be comprehended by those with a 10^th^ grade education or higher. According to the Program for the International Assessment of Adult Competencies (PIAAC), only 13% of Americans between the ages of 16 and 65 were able to perform at the highest level of proficiency on the literacy scale [4]. As per a 2003 study by the National Assessment of Adult Literacy (NAAL), approximately 36% of Americans had basic or below basic health literacy, 53% had an intermediate level, and only 12% had a proficient level of health literacy [5]. For these reasons, many sources agree upon reducing the target readability for studies. The Joint Commission recommends the 5^th^ grade level as many Medicare beneficiaries can only comprehend at that grade level [6, 7]. A manual on health literacy published by the American Medical Association recommends a readability of a 6^th^ grade level, whereas the National Institutes of Health recommends a target of 7-8^th^ grade [8, 9].

It has been reported that improving readability of patient education materials allows for comprehension by more patients [10]. In this study, we evaluate the readability of patient education materials pertaining to gastroenterology procedures: colonoscopy, flexible sigmoidoscopy, and esophagogastroduodenoscopy (EGD). According to the NIH, in 2009, approximately 7 million upper and 12 million lower endoscopies were performed with an approximate cost of $32.4 billion dollars [11]. With gastrointestinal diseases having a substantial source of morbidity, mortality, and costs, it becomes imperative to ensure that the literature provided to patients is written at a level that is understandable to them.

## 2. Methods

Google search was performed using keywords “colonoscopy,” “sigmoidoscopy,” and “esophagogastroduodenoscopy” or “EGD” with “patient information” at the end of each search term. The results from the first page were evaluated, and the first six sources for each procedure were chosen for the study as more popular and accessible websites are listed first on Google searches. Only the textual information from a total of 18 studies were saved in individual Microsoft Word Documents (Richmond, WA) from ten difference sources. Patient education materials were provided from American Society for Gastrointestinal Endoscopy (ASGE) [12], American Cancer Society (ACS) [13], National Institute of Diabetes and Digestive and Kidney Diseases (NIDDK) [14], UpToDate (Beyond the Basics) [15], WebMD [16], MedicineNet [17], Mayo Clinic [18], Patient.info [19], Society of American Gastrointestinal and Endoscopic Surgeons (SAGES) [20], and Cancer.Net [21]. Studies were then modified by removing brand names, proper names, and proprietary names as well as names of medications. Furthermore, medical terminology that was explained was also removed from the entire study prior to readability calculation. For example, if a study included the phrase “a polyp, or a small growth that is typically noncancerous and protrudes from the lining of the digestive tract, is evaluated during the procedure,” all instances of “polyp” were removed. The modifications were carried out to prevent artificial inflation of the reading grade level due to multiple instances of the same word being used but was previously explained. Finally, each study was then divided into subsections: introduction, preparation, complications, and when available, alternatives.

In total, 60 subsections were then analyzed using Readability Studio, Oleander Software Ltd. Five validated quantitative readability tests were chosen to analyze each subsection: Coleman-Liau (CL) [22], New Dale-Chall (NDC) [23], Flesch-Kincaid (FK) [24], Gunning Fog (GF) [25], and Simplified Measure of Gobbledygook (SMOG) [26]. The qualities of each test and how they derive the reading grade levels are provided in Table 1. The use of multiple scales allows for determining the complexity of the study using length of words, syllables per word, length of sentences, and use of uncommon words or in other ways. Thus, using multiple scales helps to give an average readability level. Each test displays the readability of the document as a numerical value corresponding to a grade. For example, a readability of “12” equates to the reading material that requires the education of a 12^th^ grader or high school senior to understand the material, and a readability of “14” equates to the reading material suitable for someone in the 2nd year of college. The results were further analyzed in Microsoft Excel and Prism Graphpad. A reading grade level of the 6-7^th^ grade was used as the cutoff. This was interpreted as a readability of less than 7.0 being appropriate for comprehension.

## 3. Results

**Figure 1:**
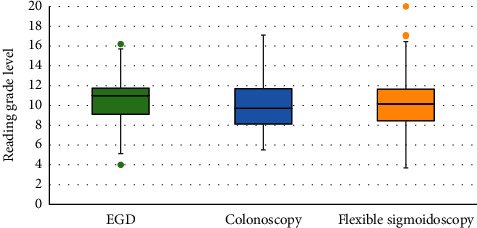
The overall reading grade distribution on a box and whisker plot for colonoscopy, flexible sigmoidoscopy, and EGD. The median for colonoscopy is 9.7 with a minimum and maximum of 5.5 and 17.1, respectively. The median for flexible sigmoidoscopy was 10.2 with a minimum and maximum of 3.7 and 20, respectively. Finally, the median for EGD was 11.0 with a minimum and maximum of 4.0 and 16.2.

**Figure 2:**
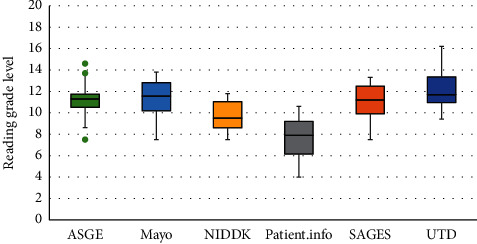
Box and whisker plot analysis of the EGD reading material. The American Society for Gastrointestinal Endoscopy (ASGE) had a median of 11.3 (minimum-maximum of 7.5–14.6), Mayo Clinic scored a median of 11.6 (7.5–13.8), the National Institute of Diabetes and Digestive and Kidney Diseases (NIDDK) scored a median of 9.5 (7.5–11.8), Patient.info had a median of 7.9 (4–10.6), the Society of American Gastrointestinal and Endoscopic Surgeons (SAGES) scored a median of 11.2 (7.5–13.3), and finally UpToDate Beyond the Basics (UTD) had scored a median of 11.7 (9.4–16.2).

**Figure 3:**
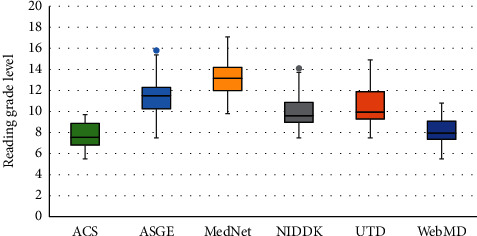
Box and whisker plot analysis of the reading material pertaining to colonoscopies. The American Cancer Society (ACS) had a median of 7.6 (5.5–9.7). The American Society for Gastrointestinal Endoscopy (ASGE) had a median of 10.3 (7.5–15.8), Medicine.Net (MedNet) had a median of 12.0 (9.8–17.1), the National Institute of Diabetes and Digestive and Kidney Diseases (NIDDK) scored a median of 9.6 (7.5–14.1), UpToDate Beyond the Basics (UTD) had scored a median of 10.0 (7.5–14.9), and finally, WebMD had a median of 8.0 (5.5–10.8).

**Figure 4:**
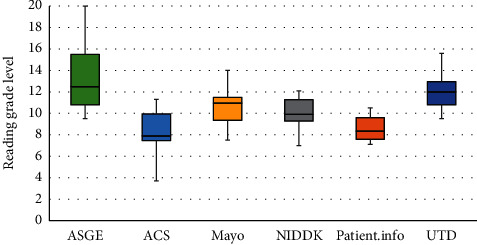
Box and whisker plot analysis of the reading material regarding flexible sigmoidoscopies. The American Society for Gastrointestinal Endoscopy (ASGE) had a median of 12.5 (9.5–20), the American Cancer Society (ACS) had a median of 7.9 (3.7–11.3), Mayo Clinic had a median of 11.0 (7.5–14.0), the National Institute of Diabetes and Digestive and Kidney Diseases (NIDDK) scored a median of 9.9 (7.0–12.1), Patient.info had a median of 8.4 (7.1–10.5), and UpToDate Beyond the Basics (UTD) had scored median of 12.0 (9.5–15.6).

**Figure 5:**
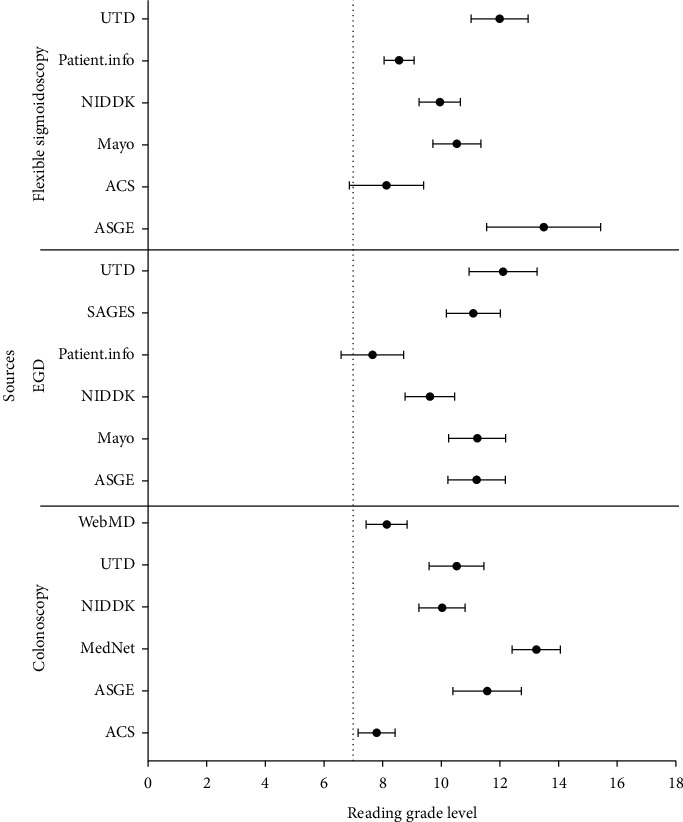
Forest plot represents the mean and 95% confidence interval of each of the procedures. With the exception of the American Cancer Society for Flexible Sigmoidoscopy and Patient.info for EGD, all studies were significantly above the recommended reading grade level of the 7^th^ grade.

Studies on colonoscopy, flexible sigmoidoscopy, and EGD had mean readability grades of 10.2, 10.3, and 10.5, respectively. Box and whisker plot analysis revealed that EGD, colonoscopy, and flexible sigmoidoscopy had median readabilities of 11.0, 9.7, and 10.2, respectively (Figure 1). Within studies pertaining to EGD, Patient.info was the easiest to comprehend with a readability of 7.9 followed by NIDDK with 9.5, SAGES with 11.2, ASGE with 11.3, Mayo Clinic with 11.6, and finally, UTD was the most difficult to comprehend with a readability of 11.7 (Figure 2). Within colonoscopy, ACS had the easiest readability with a median of 7.6, whereas the most difficult to comprehend study was that of MedicineNet, with a median of 12.0. WebMd, NIDDK, UTD, and ASGE had median readabilities of 8.0, 9.6, 10.0, and 11.5 (Figure 3). Finally, under flexible sigmoidoscopy, the easiest study was written by ACS, followed by Patient.info, NIDDK, Mayo Clinic, UTD, and ASGE with median readability of 7.9, 8.4, 9.9, 11.0, 12.0, and 12.5 respectively (Figure 4). The readability is also represented with a forest plot as a mean of each study with a 95% confidence interval (Figure 5). All values less than the vertical line are considered within the recommended range. From this representation, with the exception of two studies, all of them were significantly higher in difficulty than the recommended reading level.

**Figure 6:**
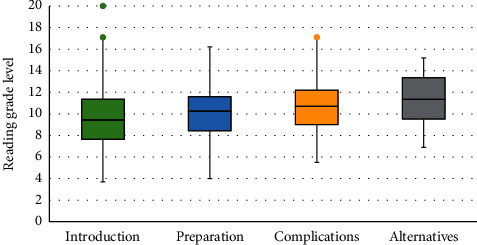
Box and whisker plot analysis of the readability of each subsection. Introduction had a median of 9.5 (3.7–20), preparation had a median of 10.3 (4–16.2), complications had a median of 10.7 (5.5–17.1), and finally alternatives had a median of 11.4 (6.9–15.2).

On further analysis of subsections, “Introduction” was the easiest to comprehend with a readability of 9.5. This was followed by “Preparation” with a readability of 10.3, “Complications” with 10.7, and “Alternatives” with 11.4 (Figure 6). It can be seen that most of the subsections do not meet the requirements of readability.

Of the 18 studies analyzed, two were in the 7^th^, 9^th^, and 12^th^ grade ranges, three were in the 8^th^ and 10^th^ grade ranges, three were in the 11^th^ grade range, two were in the 12^th^ grade range, one was at the level of a college freshman, and no studies were within the recommended range of at or below the 6-7^th^ grade level.

## 4. Discussion

### 4.1. Significance

There are significant data suggesting that providing an explanation to patients in a way that they can understand can improve health outcomes. Patients with lower health literacy have been shown to require more hospitalizations and emergency care, poor use or noncompliance with medications, and overall worse outcomes [27]. In another study concerning elderly patients, the all-cause mortality for those with inadequate health literacy was 52% higher compared to those with adequate health literacy [28]. According to a 2007 report by the Department of Health Policy at the George Washington University, it is estimated that poor health literacy can have a burden of $106–$238 billion on the national scale [29].

In a study published in Gastrointestinal Endoscopy journal, of approximately 13,000 colonoscopies, 24% had suboptimal bowel preparation [30]. This had the risk of increasing the miss rate of adenomas as well as increasing the costs of colonoscopies by 12–22%, which was roughly estimated by a 2002 study [31]. There is some evidence to suggest that bowel preparation scores can be improved with written instructions in addition to verbally teaching the patients [32]. Patient education materials have been found to be useful in improving patients' knowledge and helping with their decision-making [33]. One can argue that materials usually are of more benefits to patients if they are well-designed and written to take into account the education level of the patients as they are more likely to be compliant with them.

In our study, patient education materials were shown to be written at a level that was alarmingly too high. Even the easiest to comprehend studies were still above the recommended level. On a subsection analysis, “Complications” had a score of almost the 11^th^ grade, which means many patients may not be able to understand what complications they are often told to be vigilant for.

### 4.2. Study Design

Despite modifications to the studies that improved the readability scores, patient education materials reviewed in this study were still above the recommended 6^th^ grade level. The reason for modifying the studies was to remove the bias that comes with discussing medical literature. As made evident in Table 1, some of the formulas use syllable per word to determine readability. The word “sigmoidoscopy” has five syllables; so a study that mentions this procedure a number of times will inherently have an inflated score. For this reason, we decided to remove any terminology that was well explained without introducing new terms.

### 4.3. How to Improve Patient Education Materials

From our analysis, the studies that scored closer to the recommended grade, such as the ACS study on colonoscopy, did so because the topic was explained in a easier way to comprehend terminology and with explanations of medical terms. This study elucidates the fact that greater emphasis on clear and simple language is warranted in order to create information that is suitable for the average American.

Other ways to improve the readability is to use shorter words or words with fewer syllables when possible, for example, the use of the word “cancer” instead of “malignancy,” and use of more common words or self-descriptive terms in place of medical jargon, for example, the use of “high blood pressure” instead of “hypertension.” In addition to the use of shorter syllabic and simpler words, simplifying sentence structure by using shorter sentences to get the same point across can also help with the readability, for example, instead of

  “Pancreatitis is an inflammatory condition of the pancreas that is characterized by abdominal pain and elevated levels of pancreatic enzymes”

One can use

  “Pancreatitis is the inflammation of the pancreas. It presents with abdominal pain as well as increases in certain pancreas-related enzymes or chemicals.”

Furthermore, the use of visual aids has also been significant in improving readability and understandability [34]; however, this was not assessed in this study.

We acknowledge that adopting these practices only improve the number behind these scores. It is yet to be studied what type of impact the improvement of patient education materials has on patients. Finally, in this study, we did not measure the accuracy of each study.

## Figures and Tables

**Table 1:** The different formulas used to determine the reading grade levels of each study. An output of 6.5 would be the equivalent to someone who has finished half of their 6^th^ grade.

Test	Formula
Coleman-Liau Index	=0.0588L − 0.296S − 15.8
	L: average number of letters per 100 words.
	S: average number of sentences per 100 words
Flesch-Kincaid	=(0.39 × ASL) + (11.8 × ASW) − 15.59
	ASL: average sentence length, i.e., the number of words divided by number of sentences
	ASW: average number of syllables per word, i.e., the number of syllables divided by the number of words
New Dale-Chall	=0.1579 × PDW + 0.0496 × ASL + 3.6365
	PDW: percentage of difficult words, i.e., not commonly understood by a 4^th^ grader
Gunning Fog	=0.4 (ASL + PHW)
	PHW: percentage of hard words, i.e., with three or more syllables
SMOG	=3 + $\sqrt{\text{PSW}}$
	PSW: polysyllable word, i.e., with three or more syllables

## Data Availability

The data used to support the findings of this study are available from the corresponding author upon request.
